# Scapular Spine Stress Fracture Following Reverse Total Shoulder Arthroplasty: Successful Management with Low-Intensity Pulsed Ultrasound

**DOI:** 10.7759/cureus.10978

**Published:** 2020-10-16

**Authors:** Esther Wright, Quen Tang, Edward Ibrahim

**Affiliations:** 1 Trauma and Orthopaedics, West Middlesex University Hospital, London, GBR; 2 Surgery, Imperial College London, London, GBR

**Keywords:** glenohumeral osteoarthritis, non-union, reverse shoulder arthroplasty, scapula

## Abstract

Scapular spine stress fractures are a rare but well-recognised complication following reverse total shoulder arthroplasty (RTSA). They present a challenge with no consensus on management. Both operative fixation and conservative measures are associated with high rates of mal- or non-union and decreased functional outcomes.

We present the case of a 60-year-old female, who presented with a scapular spine fracture one year following RTSA. Treatment consisted of initial immobilisation, physiotherapy and the application of a portable low-intensity pulsed ultrasound (LIPUS) system (EXOGENÒ Ultrasound Bone Healing System, Bioventus, Durham NC, Netherlands). Following a three-month treatment course, there was a significant improvement in patient-reported pain and functional scores (Oxford Shoulder Score from 5/48 to 38/48). Sequential radiographic imaging confirmed fracture union. Clinicians may consider the use of LIPUS therapy as a potential adjunctive treatment modality to promote the union of scapular spine stress fractures following RTSA.

## Introduction

In 1993, Grammont and Baulot pioneered the current concepts of reverse total shoulder arthroplasty (RTSA) [1]. The design principles allowed the shoulder to elevate in the absence of a superior cuff and rapidly became a reliable option for patients with rotator cuff arthropathy. The indications for RTSA have since extended and include an irreparable rotator cuff in the elderly, primary osteoarthritis with deformed glenoid, revision of failed arthroplasty and complex proximal humerus fractures [2-3].

RTSAs are associated with a number of postoperative complications, including instability, infection, glenoid and humeral loosening, scapular notching, acromion and scapular spine stress fractures and peripheral nerve injuries [2,4]. Fundamental to the design principles, restoration of appropriate deltoid tension is key to optimising function [5]. Over-tensioning can inadvertently increase the stresses across the scapular spine, leading to insufficiency fractures. Further reported causes of fractures include the placement of a superior metaglene screw and patient factors contributing to low bone mineral density, highlighting the multifactorial nature of this rare complication [6]. Incidence following RTSA is reported between 0.8% and 7.2% [5]. Although infrequent, scapular spine stress fractures present a challenging complication associated with poor outcomes [5,7].

Both conservative and surgical fixation of scapular spine stress fractures have been described with unpredictable outcomes. Consequently, consensus on management is not well-defined [5,8-9]. Conservative measures include a period of initial immobilisation, analgesia and subsequent physiotherapy. The use of low-intensity pulsed ultrasound (LIPUS), however, is not, to our knowledge, been reported in the literature. A systematic review demonstrates that LIPUS successfully reduces time to radiographic union in other types of fracture [10]. The National Institute for Health and Care Excellence (NICE) has approved LIPUS (EXOGENÒ Ultrasound Bone Healing System, Bioventus, Durham NC, Netherlands) to treat non-union in long bone fractures, estimating savings of approximately £2,407 per patient as compared with operative management. The cost-effectiveness for scapular spine fractures has not yet been established [11]. We present a case of scapular spine stress fracture following RTSA, which was successfully managed non-operatively using LIPUS.

## Case presentation

A 60-year-old female, with a diagnosis of left-sided glenohumeral osteoarthritis, presented in February 2018 with a two-year history of worsening pain, weakness and stiffness in the affected shoulder. These symptoms had a profound effect on her activities of daily living (ADLs), leading to a deterioration in mental health and a requirement for regular analgesia. Past medical history included a gastric bypass, a lower limb deep vein thrombosis, for which she had completed treatment, bilateral carpal tunnel decompression and bilateral osteoarthritis of the hands. She was a non-smoker, did not drink alcohol and mobilised with a stick indoors.

Initial physical examination revealed posterior joint line tenderness and both limited active anterior elevation (40°) and active external rotation (10°). There was no evidence of rotator cuff pathology or neurological deficit of the forearm or cervical spine. Her computed tomography (CT) revealed significant glenoid retroversion and posterior static subluxation of the humeral head.

**Figure 1:**
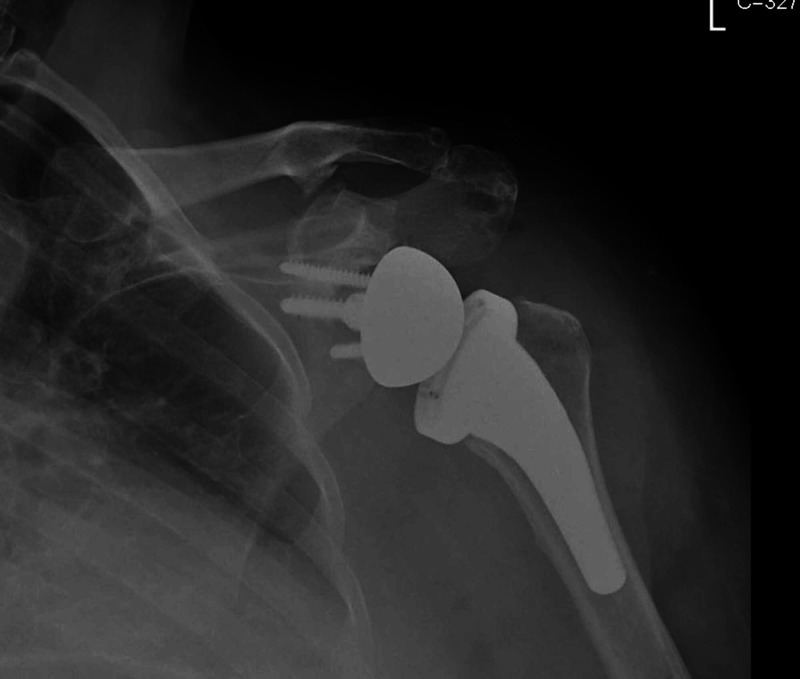
Initial post-operative plain radiograph of the left shoulder (AP view) AP: anteroposterior

**Figure 2:**
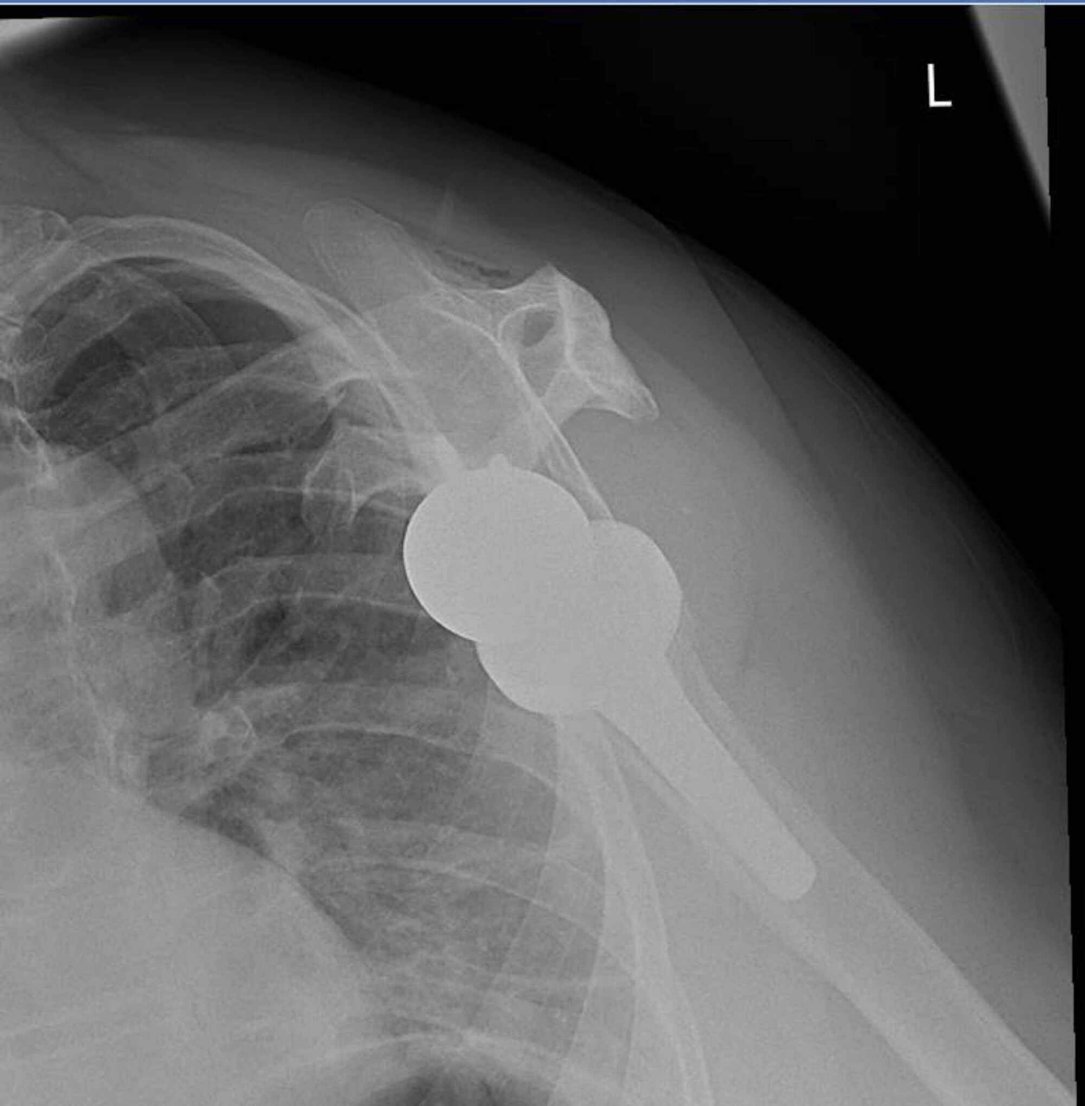
Initial post-operative plain radiograph of the left shoulder (Y-view)

Following the implantation of RTSA, her initial post-operative recovery was uncomplicated. Figures 1-2 demonstrate initial post-operative plain radiographs demonstrating satisfactory implant positioning. Five months later, she presented with acute-onset severe posterosuperior shoulder pain with no precipitating trauma. Despite an absence of acute pathology on plain radiographs or CT scan, her symptoms remained persistent.

**Figure 3:**
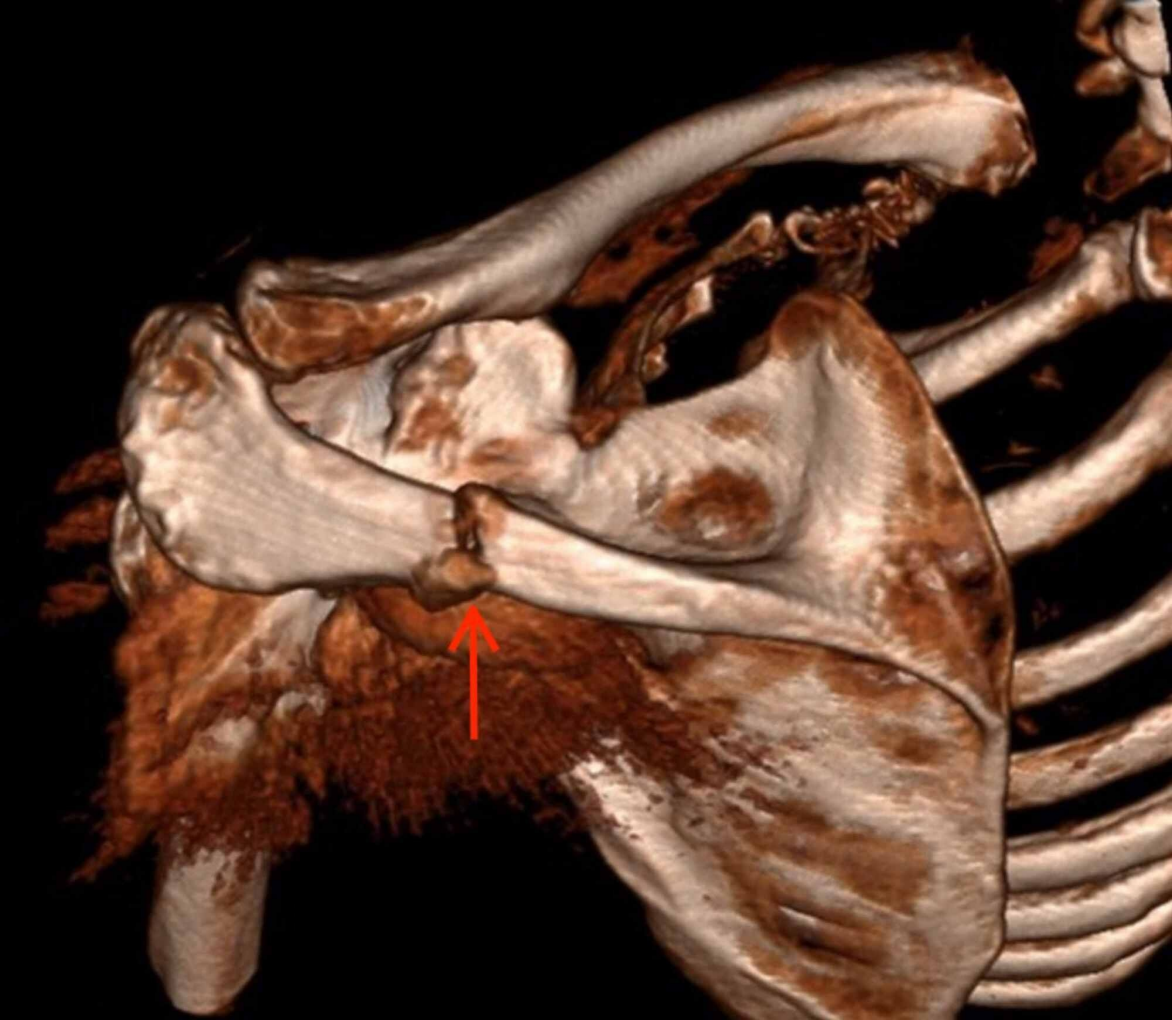
3D re-format CT scan demonstrated a fracture of the medial third of the left scapula spine CT: computed tomography

**Figure 4:**
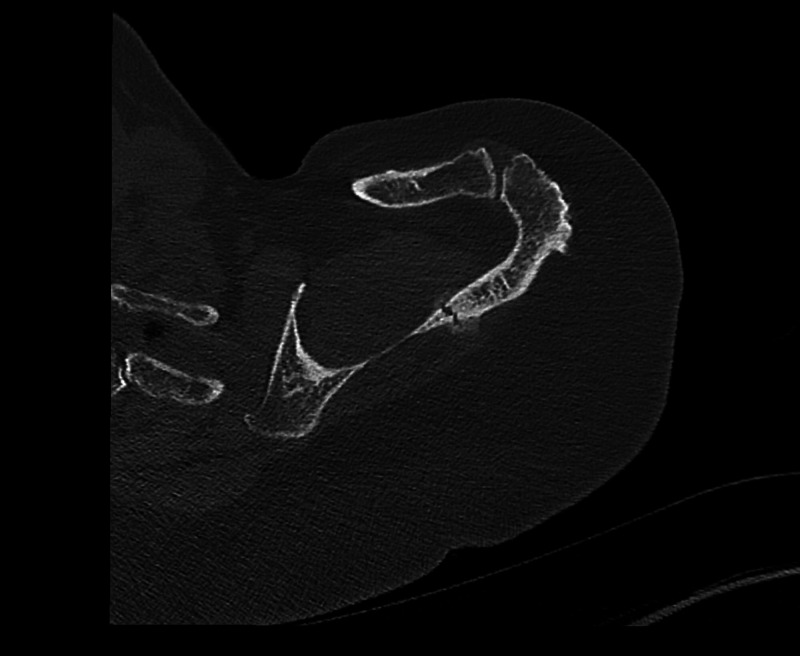
Axial section demonstrating a scapular spine fracture prior to EXOGENÒ therapy EXOGENÒ Ultrasound Bone Healing System, Bioventus, Durham NC, Netherlands

A repeat CT scan performed 12 months post-operatively demonstrated an undisplaced fracture of the middle third of the scapula spine, without extension to the implant (Figure 3 and Figure 4). There was no evidence of coracoid fracture, hardware failure, loosening or infection.

LIPUS therapy

After initial advice to rest the left shoulder using a broad arm sling, the patient was provided with a portable LIPUS system (EXOGENÒ Ultrasound Bone Healing System), which was used every day for a three-month period. Daily application directly over the fracture site was conducted for a period of 20 minutes as per manufacturer guidelines [10]. The patient achieved 100% compliance over three months. Active physiotherapy exercises were gradually re-instituted as her symptoms subsided.

Outcome

**Figure 5:**
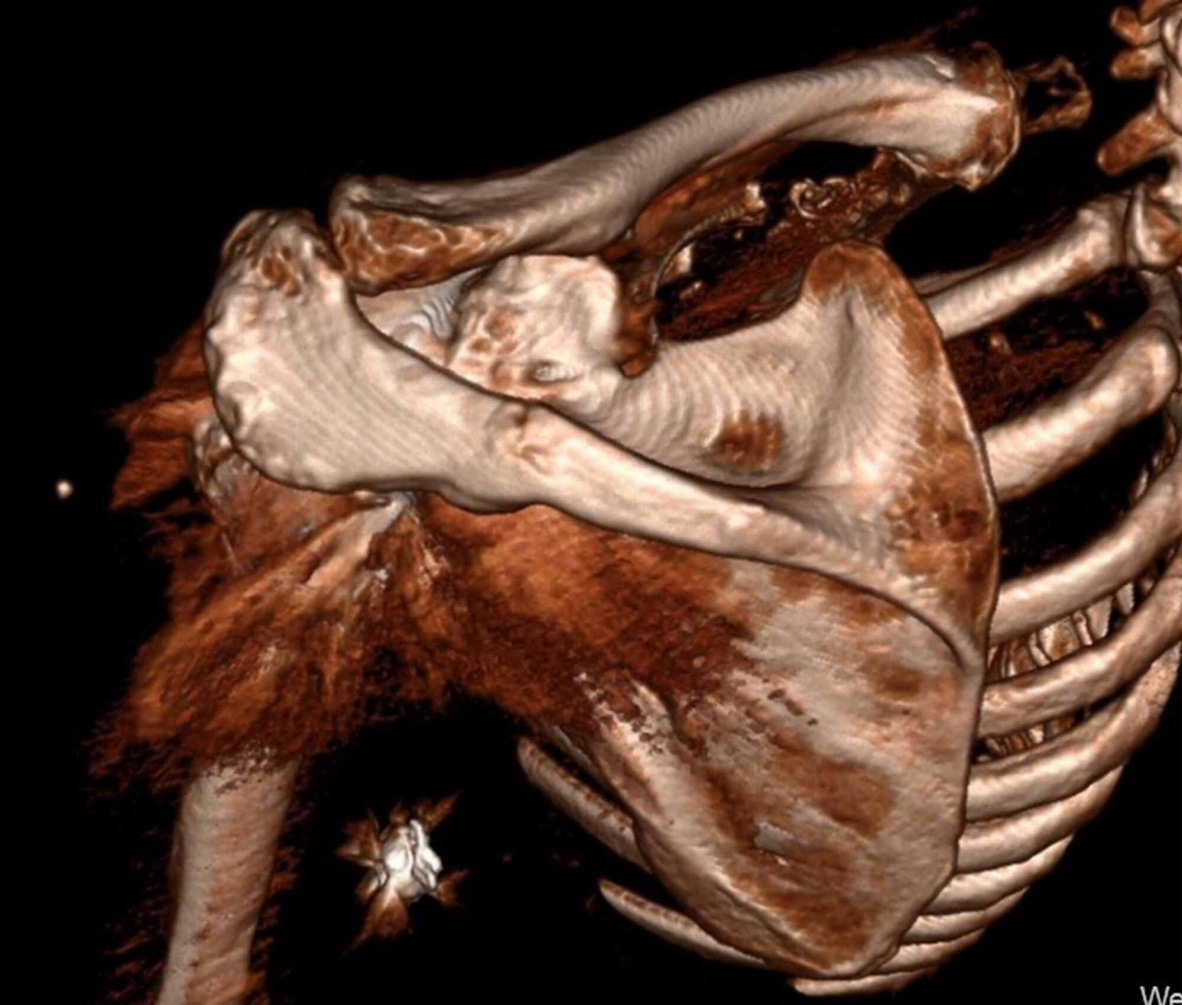
3D re-format CT scan taken three months later demonstrates fracture resolution CT: computed tomography

**Figure 6:**
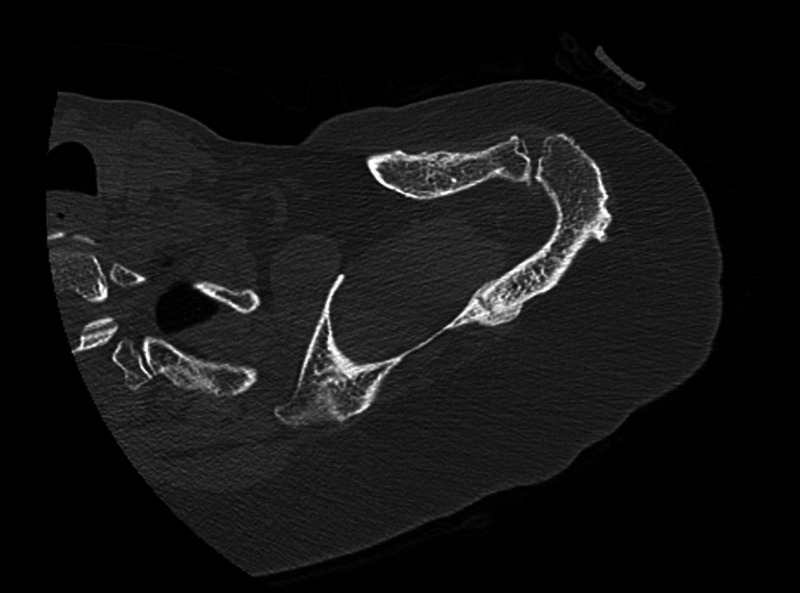
Axial section demonstrating a healed scapular spine fracture following EXOGENÒ therapy EXOGENÒ Ultrasound Bone Healing System, Bioventus, Durham NC, Netherlands

The patient reported a rapid resolution of pain within a few weeks of initiating LIPUS therapy. Following the completion of treatment, her posterosuperior shoulder pain had completely resolved. Her Oxford Shoulder Score (OSS) also improved from 5/48 at the time of stress fracture diagnosis to 38/48 at the point of LIPUS therapy completion. This was paralleled by an improvement in her range of active anterior elevation and active external rotation from 30° and 0°, respectively, prior to commencing treatment, to 130° and 30° respectively at completion. Restoration of the patient’s ability to perform unassisted ADLs was also achieved (dressing and performing overhead activities). Despite this complication, the patient reported overall satisfaction greater than her pre-operative status, which is in keeping with current literature [11]. Repeat imaging demonstrated bony union with substantial surrounding callous formation (Figure 5 and Figure 6).

## Discussion

The annual incidence of RTSA is increasing within Europe and the United States, highlighting the importance of successfully recognising and managing potential complications [3]. Scapular spine stress fractures are particularly challenging due to universally poor outcomes and poorly defined treatment strategies [12]. Levy et al. classified post-RTSA scapular stress fractures into three types, of which Type III fractures include the scapular spine [13]. Risk factors for Type III fractures are broadly classified into patient or operative factors. Osteoporosis is considered the most significant patient risk factor, with limited evidence for other contributors, such as alcohol excess, smoking and autoimmune disease, all of which are associated with low bone mineral density [12]. Operative factors include the placement of a superior metaglene screw, which may act as a stress riser, and excessive deltoid tensioning, although the evidence for both is largely conflicting [8,14-15].

Type III fractures may present late and are often undetected on plain radiographs. The mean time to diagnosis is reported as 15.2 months from a retrospective multicentre analysis of 53 patients [14]. This is consistent with our case, in which the patient presented with acute pain four months post-operatively, with no definitive evidence of radiographic fracture for a further eight months. This may be explained by an initial stress reaction progressing to an undisplaced and finally displaced fracture [12]. Plain radiographs are unreliable in identifying undisplaced fractures and a high index of suspicion should be maintained if symptoms persist [5,8,13]. Otto et al. reported that 32.1% of patients with persistent pain and normal post-operative plain radiographs were later diagnosed with displaced stress fractures [14]. Early and repeated cross-sectional imaging of the entire scapula is, therefore, recommended in cases of suspected fracture [12].

Numerous approaches in the management of Type III fractures have yielded consistently poor results [5,8,14,16]. Of four patients in a small case series, who were managed with open reduction and internal fixation, one proceeded to revision surgery and another required metalwork removal. Union rates were not reported [8]. Conversely, another series of nine cases, all managed non-operatively, reported eight incidences of non-union at 12 months. Overall, however, reported functional outcomes were improved compared to pre-operative status [16]. A 50% non-union rate was also reported in four patients with scapular spine stress fractures treated with sling immobilisation [17]. This figure was reproduced by Teusink et al. [5]. Walch et al. reported two cases of fracture union following non-operative management at 24 months [7]. Despite the majority of studies favouring non-operative treatment, the outcomes are unpredictable and optimal management is not defined.

## Conclusions

We report a case of a scapular spine stress fracture following RTSA, where a rapid and significant improvement in symptoms, function and fracture union was achieved through non-operative management. It is unclear whether LIPUS therapy has a direct positive effect on the bony union rates of scapular spine stress fractures following RTSA. Nonetheless, given the speed of resolution in this case, in comparison to the poor union rates reported in the literature despite invasive and prolonged treatment, we would suggest consideration of LIPUS therapy as a potential adjunctive non-invasive treatment modality. In order to establish the reliability and effectiveness of LIPUS in promoting union for this rare complication, further research within larger patient groups must be conducted.
